# Supplementary material for: Cis-regulatory effect of HPV integration is constrained by host chromatin architecture in cervical cancers
Source: Mol Oncol. Author manuscript; Available in PMC 2024 May 9. (PMC11076994; doi:10.1002/1878-0261.13559)
Supplement: Supplementary figures [file EMS193052-supplement-Supplementary_figures.pdf]

Supplementary Figures

Supplementary Figure 1

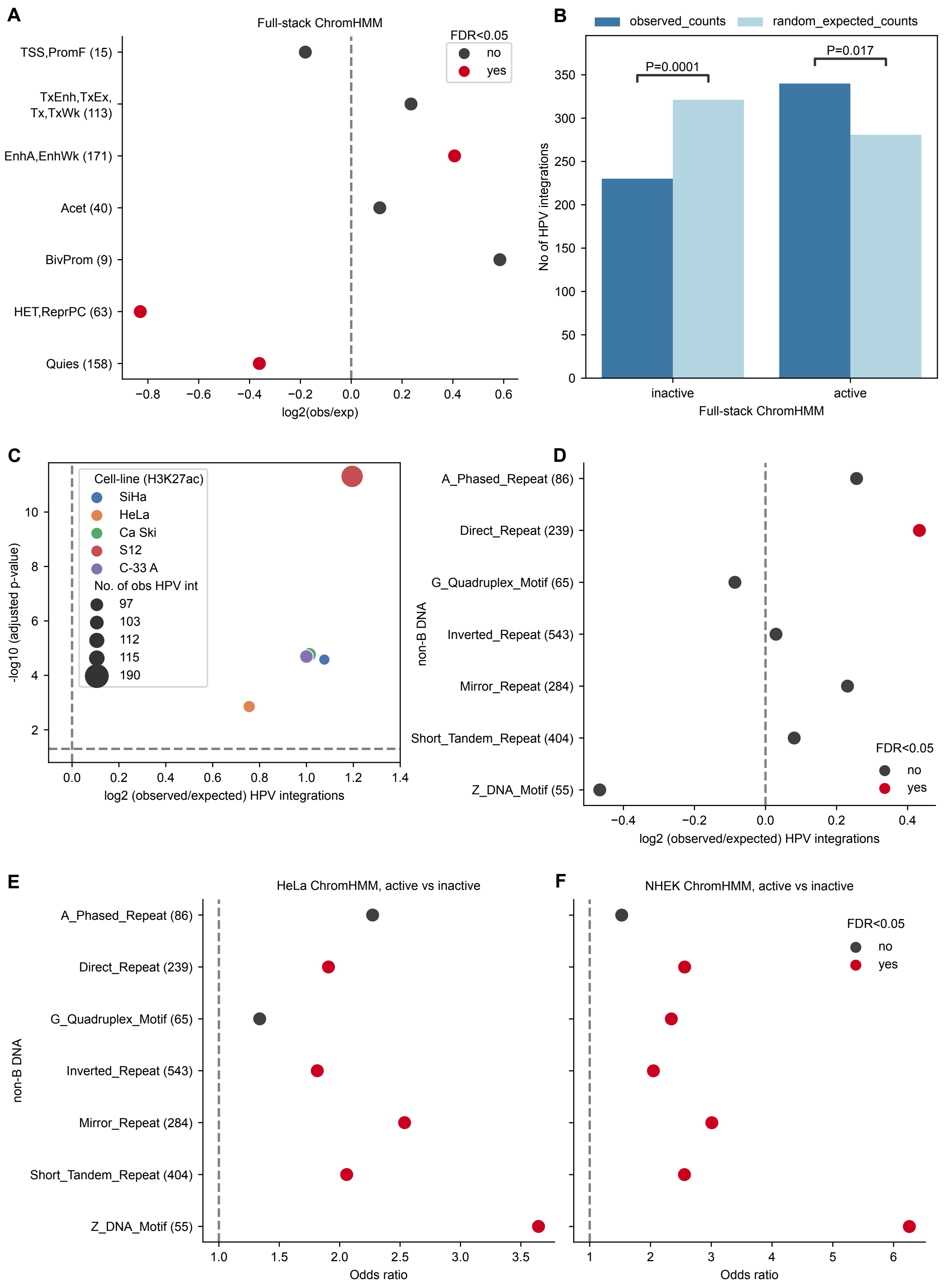

Supplementary Figure 1: Enrichment of HPV integrations in full-stack ChromHMM and non-B form DNA regions.

A) Enrichment of HPV integrations in the full-stack ChromHMM annotated regions. The x-axis represents the log2 of observed/expected number of HPV integrations. The y-axis represents the different annotations and the observed number of HPV integrations overlapping them (given in the bracket). The p-values were computed using Chi-squared goodness-of-fit test followed by FDR correction. The colour of the dots indicates whether the adjusted p-value is below the significance level of 5% or not.

B) Bar plot showing the frequency of observed and expected integrations in the active and inactive regions defined by combining full-stack ChromHMM annotations (see Methods). The p-value was calculated using a one-sample Chi-squared test.

C) Enrichment of HPV integration in active histone modification regions (H3K27ac) from cervical cancer cell lines. The x-axis represents the log2 of observed/expected number of HPV integrations and the y-axis represents the negative log10 of adjusted p-value (Chi-squared test followed by FDR correction). The horizontal dashed line represents FDR cut-off of 5%. The colour and size of the dots represent the cell line and the number of observed HPV integrations, respectively.

D) Enrichment of HPV integrations in different non-B forms of DNA predicted genome-wide. The x-axis represents the log2 of observed/expected number of HPV integrations. The y-axis represents the different non-B DNA forms and the observed number of HPV integrations overlapping them (given in the bracket). The p-value was computed using Chi-squared test followed by FDR correction. The colour of the dots indicates whether the adjusted p-value is below the significance level of 5% or not.

E-F) The enrichment of HPV integrations overlapping each of the non-B forms DNA in active versus inactive regions from HeLa **(E)** and NHEK **(F)** cell lines. The x-axis represents the odds ratio calculated as the ratio of observed versus expected HPV integrations in the active region divided by the ratio of observed versus expected HPV integrations in the inactive region. The p-value was calculated using Fisher's exact test followed by FDR correction. The colour of the dots indicates whether the adjusted p-value is below the significance level of 5% or not.

Supplementary Figure 2

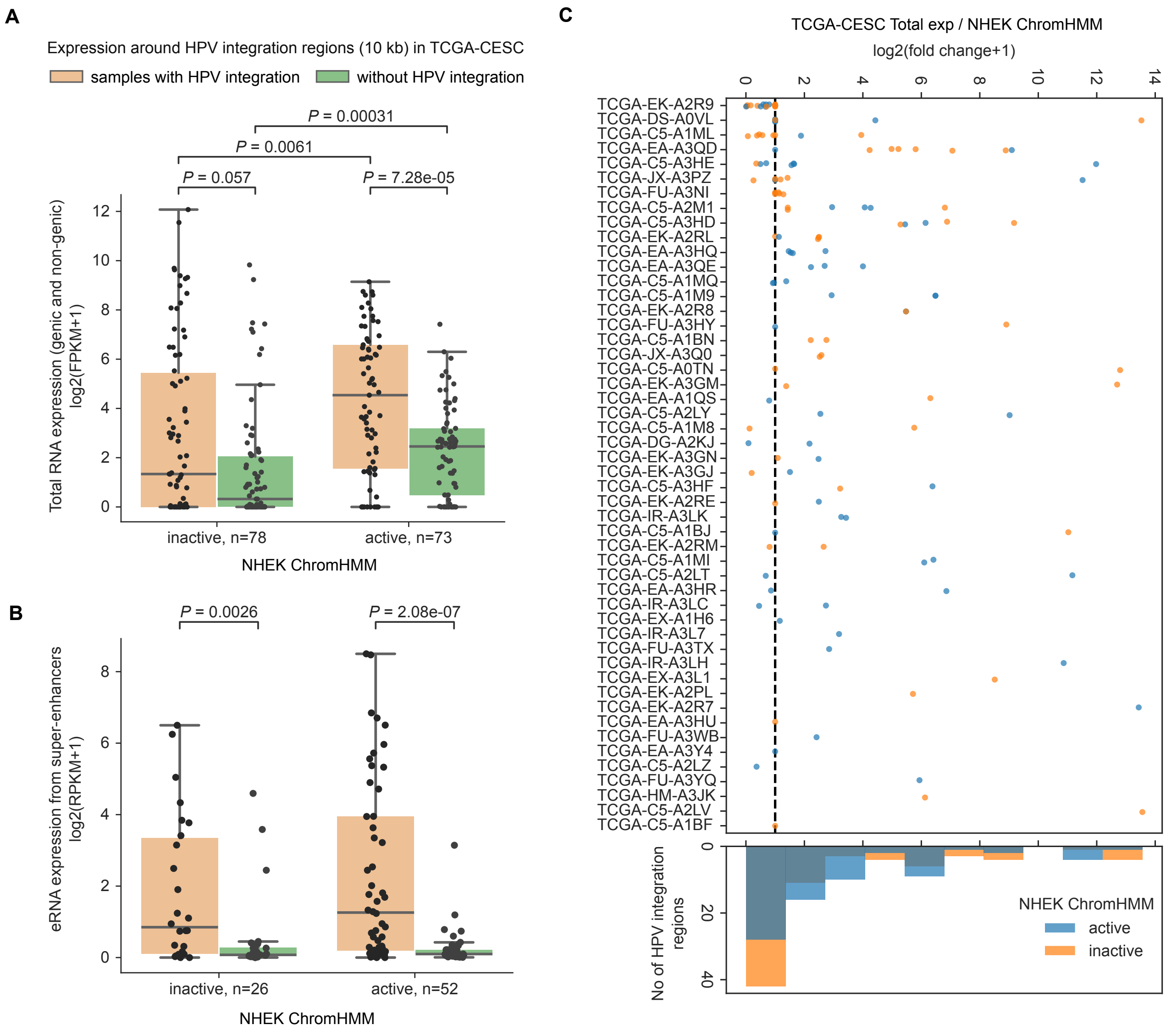

Supplementary Figure 2: Enhanced transcriptional activity near HPV integration in the context of chromatin states from NHEK.

A) Boxplot showing the total expression in the 10 kb flanking region around the HPV integration regions as compared to mean expression from TCGA-CESC samples without HPV integration in the same region. In the boxplot, the horizontal middle line indicates the median, the height of the shaded box indicates the interquartile range (IQR) and the whiskers indicate 1.5 × IQR. The x-axis represents whether the HPV integration is located in inactive (n=78) or active (n=73) chromatin region with respect to NHEK ChromHMM. The p-values were computed using Mann-Whitney U test (two-sided).

B) Same as **(A)** but for the eRNA expression from super enhancers within 10 kb on either side of the HPV integration regions located in inactive (n=26) or active chromatin regions (n=52).

C) Expression fold change associated with each of the HPV integration regions. The x-axis represents the log2 fold change, which was calculated as the total expression in the 10 kb flanking region around HPV integration regions divided by the mean expression from other samples without

HPV integration in the same genomic region. The y-axis represents the individual sample-id of TCGA-CESC samples. The colour of the dots indicates if the integration overlaps an active or inactive ChromHMM region of NHEK. The black vertical line represents the value of log2(fc+1)=1. The histogram at the bottom shows the frequency of integration regions at different fold-change bins.
Each dot in **(A-C)** represents a HPV integration region from a sample.

Supplementary Figure 3

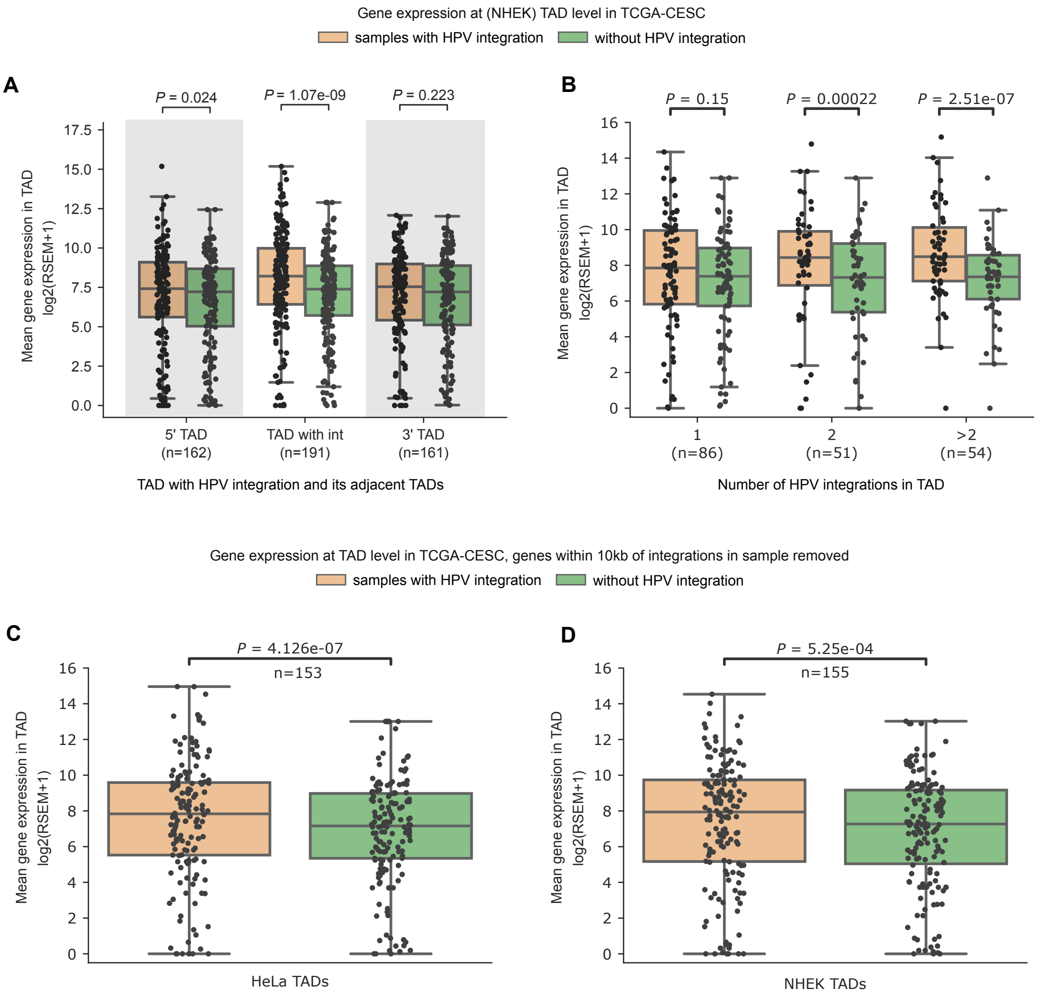

Supplementary Figure 3: HPV integration associated host gene overexpression with respect to HeLa and NHEK TAD domains.

A) TAD level gene expression in the TCGA-CESC samples with HPV integration compared to the mean expression from the samples without HPV integration in the same TADs (n=191), also for the neighbouring upstream (5’, n=162) and downstream (3’, n=161) TADs.

B) TAD level gene expression in the TCGA-CESC samples with HPV integration, separated by whether the TAD had one (n=86), two (n=51) or more than two (n=54) integrations compared to the mean expression from the samples without HPV integration in the same TADs. The TAD information was obtained from the NHEK cell line for **(A-B)**.

C-D) HeLa (n=153) **(C)** and NHEK (n=155) **(D)** TAD level gene expression in the TCGA-CESC samples with HPV integration compared to the mean expression from the samples without HPV integration in the same TADs after removing genes which are within 10 kb region of integrations in the sample.

The p-values shown in **(A-D)** were computed using the Wilcoxon signed-rank test (two-sided). In each boxplot, the horizontal middle line indicates the median, the height of the shaded box indicates the interquartile range (IQR) and the whiskers indicate 1.5 × IQR.

Supplementary Figure 4

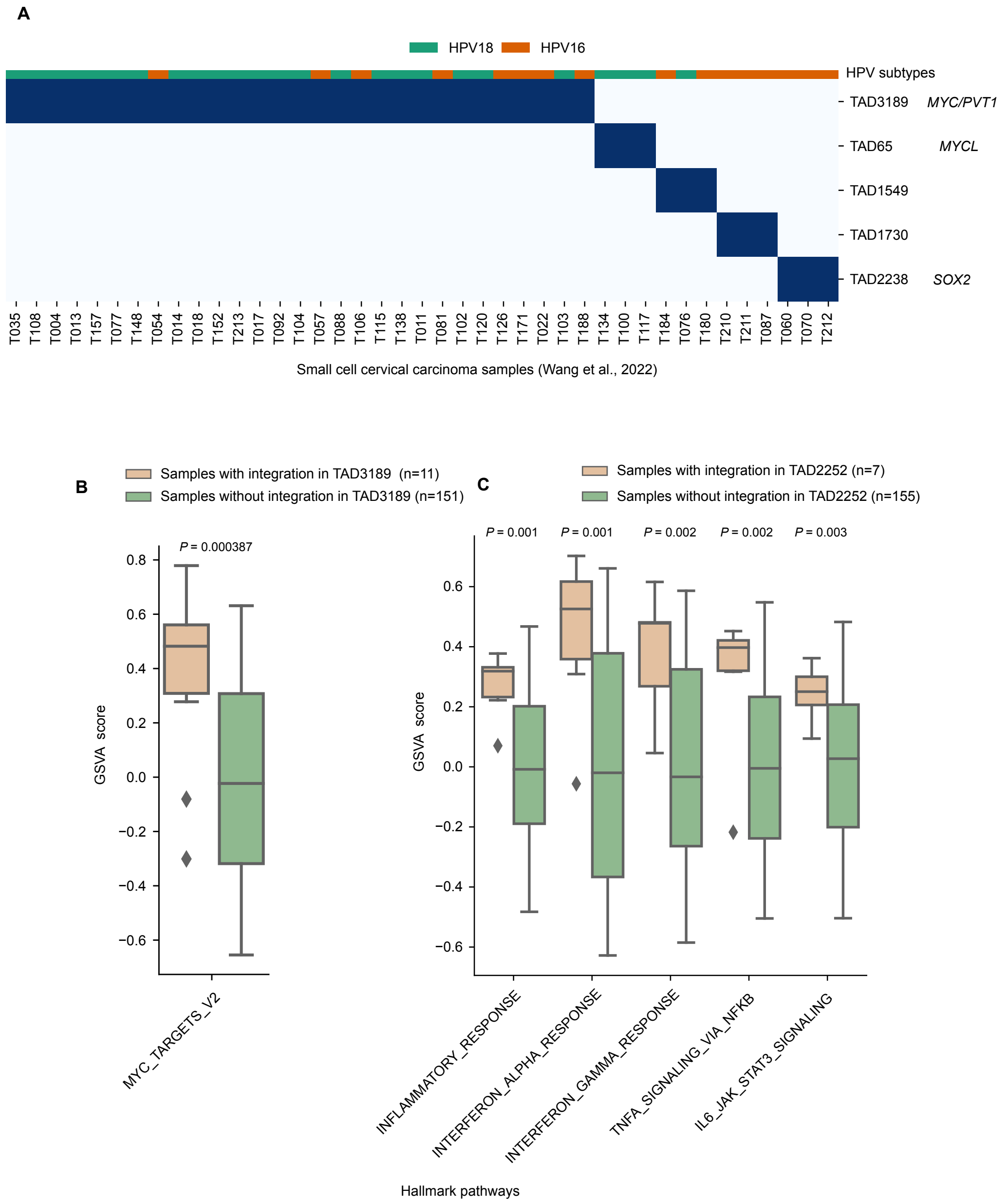

Supplementary Figure 4: Recurrently integrated TADs in small cell cervical cancer and the pathway level dysregulation in TADs with integration.

A) Heatmap shows the HeLa TADs with recurrent HPV integrations in small cell cervical cancers obtained from Wang et al., 2022[21]. The x-axis represents the sample-id and y-axis represents the TADs (denoted with distinct numbers to differentiate each TAD domain). The cancer genes identified in each TAD are listed on the right. Blue box indicates HPV integration in a particular TAD, and in a particular sample. The top row represents the HPV subtype in each of the samples.

B) The plot depicts the hallmark gene sets from MSigDB that showed significant upregulation in TCGA-CESC samples with integration (n=11) as compared to samples without integration (n=151) in TAD3189

(C) Same as (B) but for TAD2252 (samples with integration, n=7; samples without integration, n=155). In B-C, the p-values shown at the top were computed using a one-sided Mann-Whitney U test. In the boxplot, the horizontal middle line indicates the median, the height of the shaded box indicates the interquartile range (IQR) and the whiskers indicate 1.5 × IQR.

Supplementary Figure 5

A

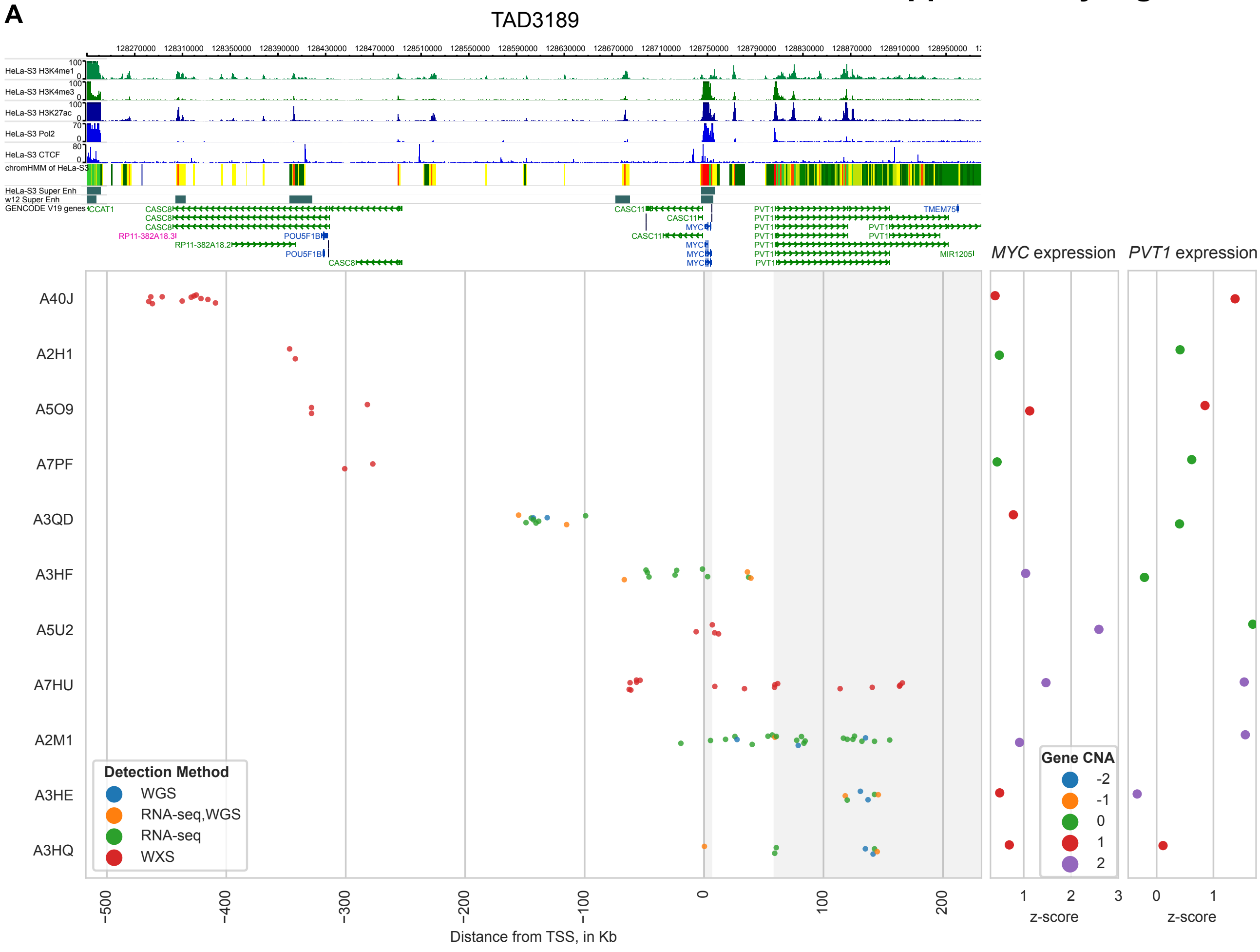

B

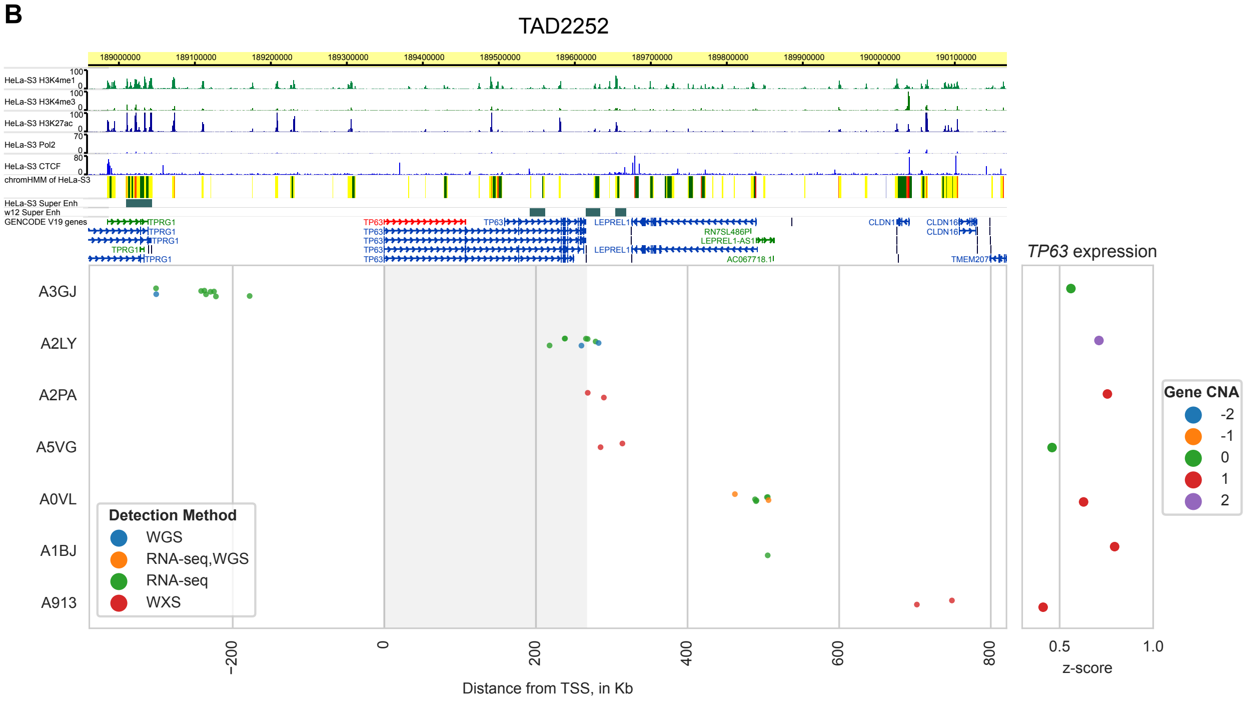

Supplementary Figure 5: Relationship between distance of HPV integration and the expression of oncogenes.

A-B) Figure shows all the TCGA-CESC samples with integration in **(A)** TAD3189 and in **(B)** TAD2252. Each of the rows indicates a separate sample with integration (detection method colour coded) in the respective TADs. The MYC and PVT1 gene coordinates falling in TAD3189, and the TP63 gene coordinates falling in TAD2252, are highlighted (in grey). The values on the x axis represent the distance from the promoter of MYC in **(A)** and promoter of TP63 in **(B)**. For each of the samples, the gene expression was represented as z-score on the right-side, and the dots were coloured with respect to relative copy number status of the gene (-2 deep deletion, -1 deletion, 0 copy neutral, 1 amplification, 2 high amplification).

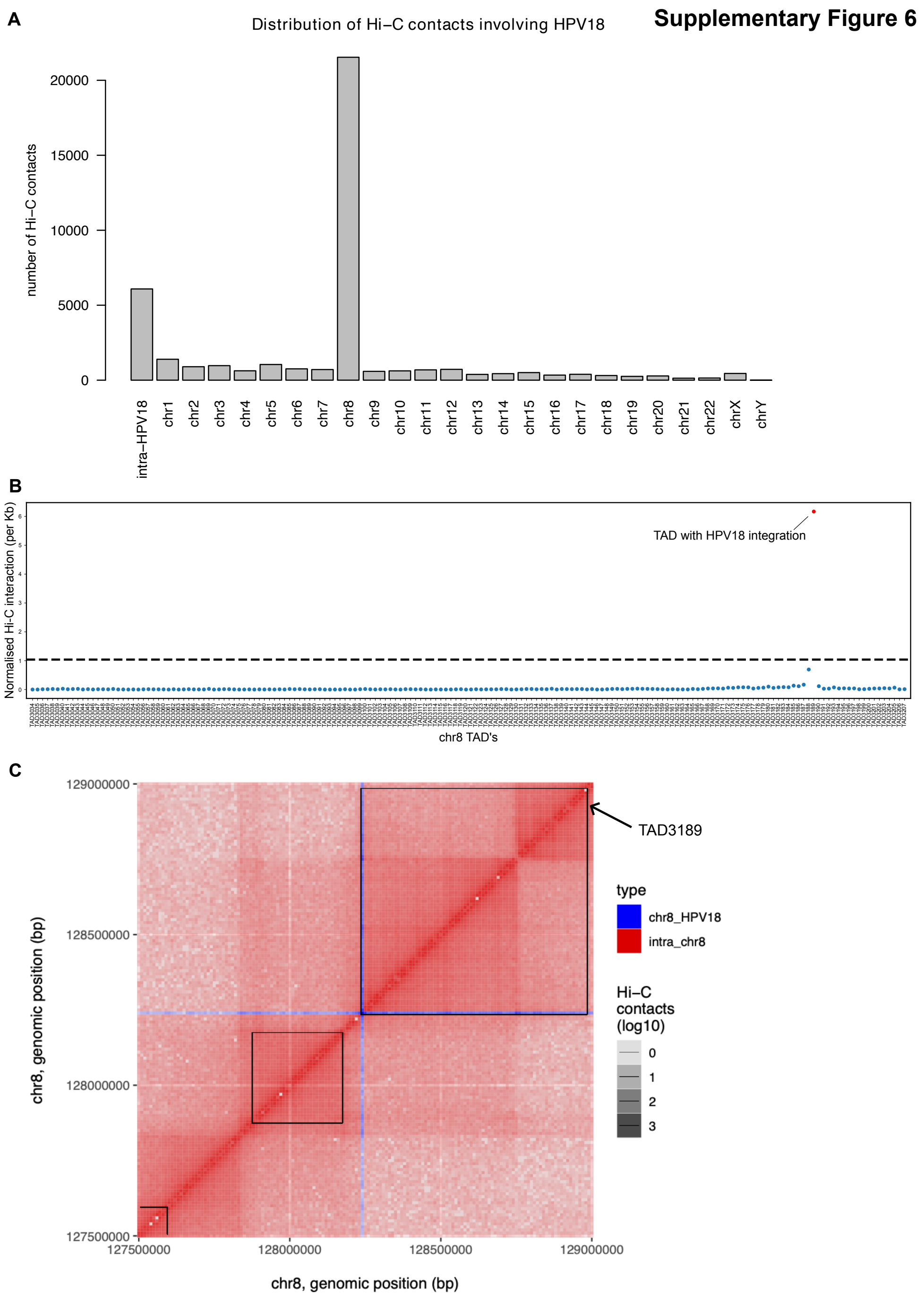

Supplementary Figure 6: TAD level interaction frequency between HPV18 integrated DNA and host genome in HeLa.

A) Bar plot shows the number of Hi-C contacts between each of the chromosomes and the integrated HPV18 genome in HeLa, and also intra-HPV18 contacts.

B) Scatterplot shows the normalised Hi-C interaction frequency per kb for all the TADs on chromosome 8 with integrated HPV18. All the TADs are arranged in a linear manner and TAD3189 with HPV18 integration is marked in red.

C) The heatmap shows the normalised Hi-C contact frequency between integrated HPV18 DNA and chromosome 8 (blue) and intra-chromosome 8 (red) regions. The black square on the top of the heatmap highlights the TADs. The TAD3189 encompasses the majority of interaction between integrated HPV18 DNA and chromosome 8.

Supplementary Figure 7

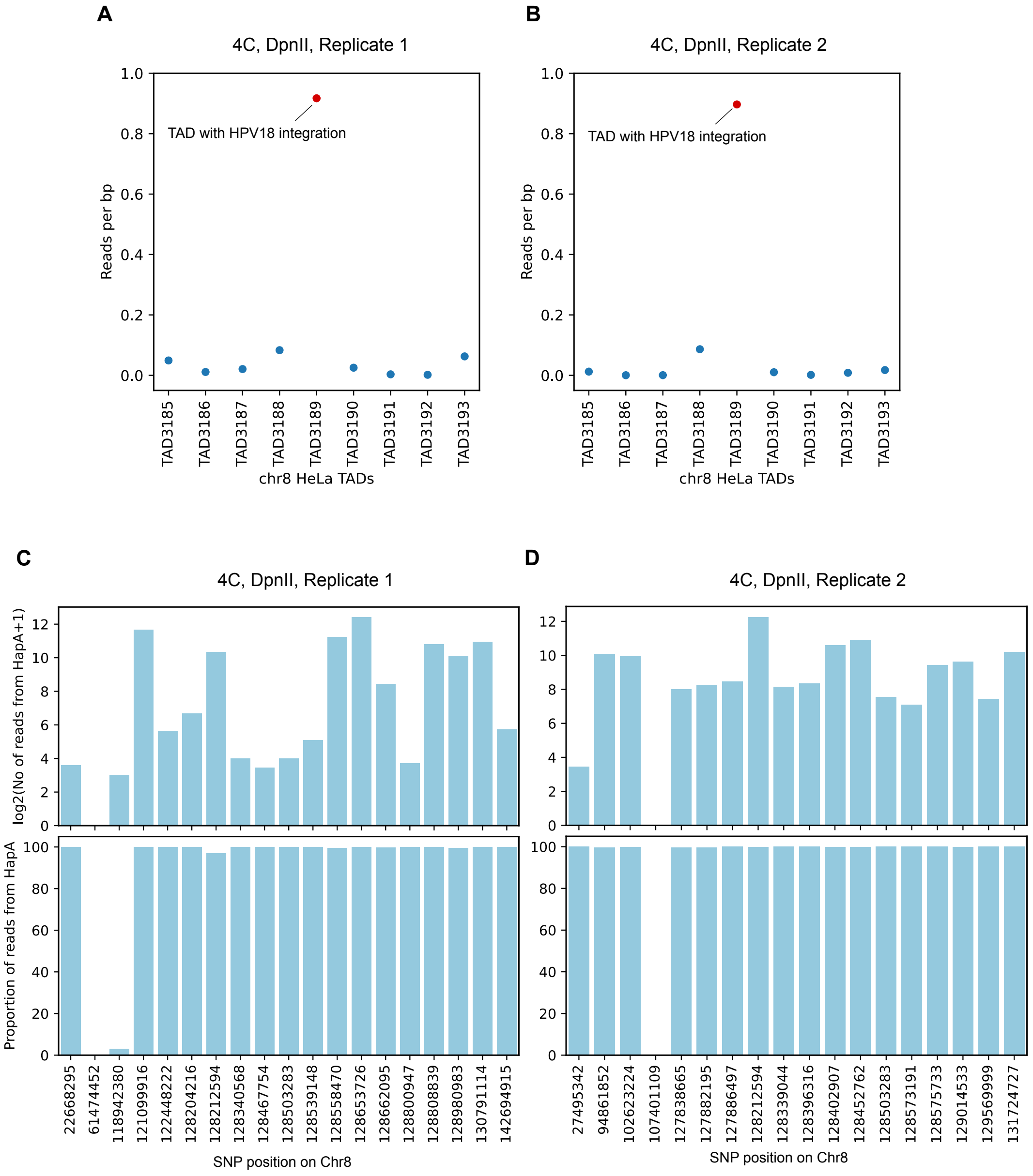

Supplementary Figure 7: 4C-seq analysis reveals haplotype-specific chromatin interactions mediated by integrated HPV18 DNA in HeLa.

A-B) Scatter plot shows the reads per bp coverage at TAD level, from the 4C-seq experiment for each of the replicates, for TAD3189 and 4 upstream and 4 downstream TADs.

C-D) Upper bar plot shows the 4C-seq read coverage for the Haplotype A at the heterozygous SNPs which overlaps the 4C-seq coverage regions and lower bar plot shows the proportion of 4C-seq reads mapping to Haplotype A at each of these heterozygous SNPs, for each of the replicates.

Supplementary Figure 8

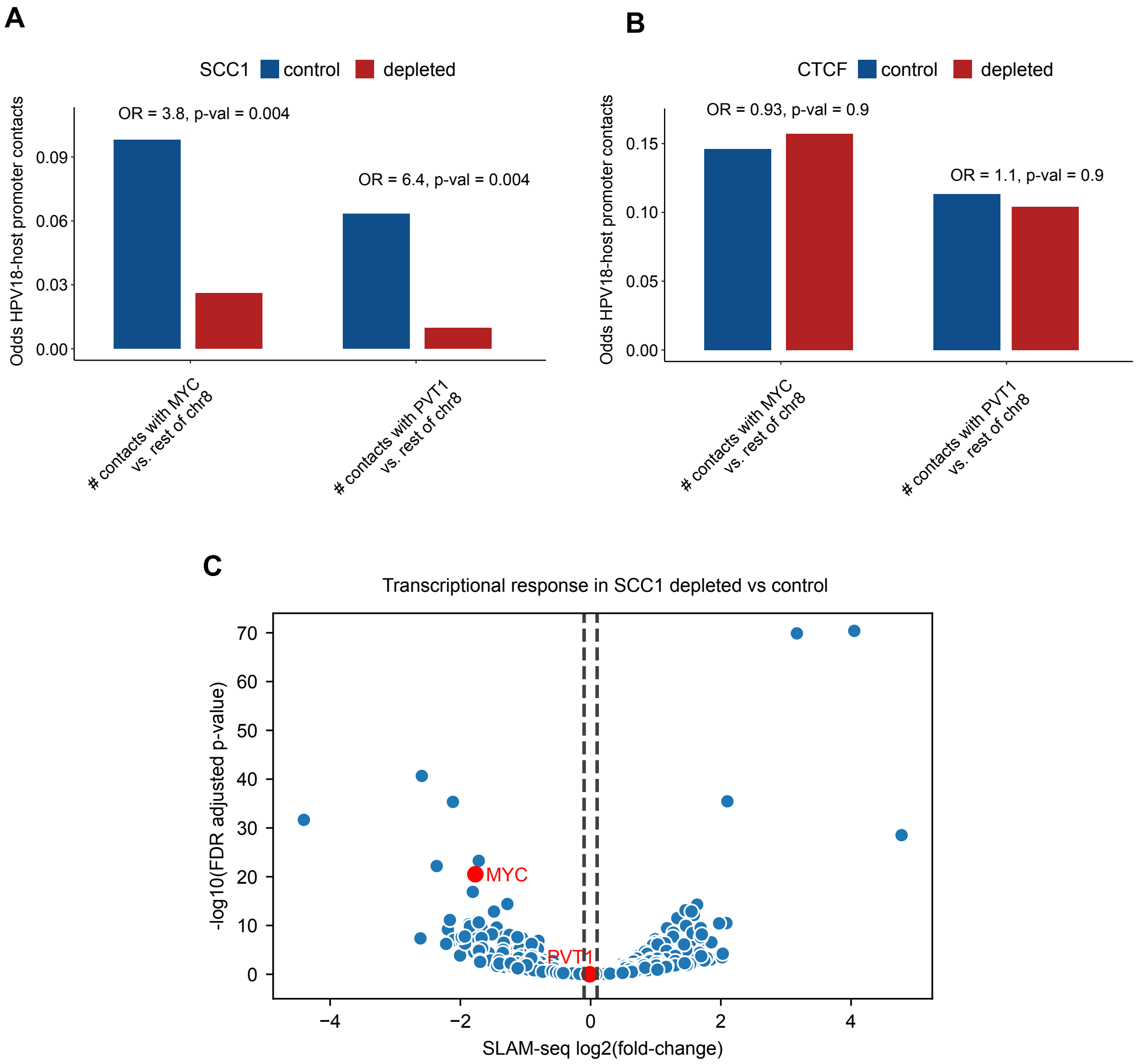

Supplementary Figure 8: Promoter Capture Hi-C analysis in HeLa depleted for Cohesin and CTCF.

A) Number of chromatin interactions between integrated HPV DNA and promoter regions of MYC/PVT1 (normalised by the number of interactions between integrated HPV DNA to the rest of chromosome 8, represented as Odds in y-axis) in SCC1 (cohesin) control and depleted condition. **B)** same as **(A)**, but for CTCF control and depleted condition. The p-values shown in panel **(A-B)** were calculated using Fisher's exact test.

C) Transcriptional response (captured using SLAM-seq) upon SCC1 depletion versus control condition. The x-axis represents the log2 fold change and the y-axis represents FDR adjusted p-value (computed using DEseq2). The vertical dotted lines indicate log2 fold-change of 0.1.
